# Internal Administration of Chrysarobin for Infantile Eczema

**Published:** 1886-08

**Authors:** 


INTERNAL ADMINISTRATION OF CHRYSAROBIN FOR INFANTILE ECZEMA —Stoeguart reports several cases of infantile eczema treated by small doses of chrysarobin. It is given from a thirteenth to a tenth or even a grain daily. The periods of cure did not exceed ten days. Theoretically, the drug is supposed to exert a constricting action on the capillaries of the skin.—*N. Y. Medical Journal.*

---

THE mortality thus far among the patients inoculated by Pasteur's method is ¾ of 1 per cent. Formerly, the mortality amounted to 16 per cent. The mortality among Pasteur's patients bitten by wolves have been 14 per cent. According to Bronardel, the mortality among wolf-bitten patients, previous to the introduction of Pasteur's methods, was 65 per cent.

Dr. Hun Su, of Pekin, China, recommends the following for uncomplicated typhoid fever:

℞ Three inches of dried umbilical cord.
One fried snake skin.
One fresh tom-cat's head.

M. Boil in five pints of water for two hours, and strain.

Sig. Tablespoonful every four hours.

---

Prof. Antonio Ceci, of Genoa, has recently extirpated the spleen successfully. The patient was a servant girl, seventeen years of age. The spleen was enlarged to such an extent that it constituted one-fifteenth the entire weight of the body. This is the seventh splenectomy performed in Italy, and is the second successful case.

---

The discovery of the alkaloid cocaine was made by Pizzi, in Bolivia, in 1857, and not by the German, Niemann, as is usually supposed. Foreth, the professor of chemistry in the University of La Paz, Bolivia, has the manuscripts left by Pizzi in his possession, in which the method of isolating cocaine is described.

---

Dr. V. Mott, who introduced the Pasteur system of preventive inoculations for hydrophobia, was obliged to discontinue the treatment in the first case operated on in the new laboratory. The patient, a boy, had attacks of fever and delirium after each inoculation.

---

Dr. W. Slayter publishes in the *Lancet* a report of a case of delirium tremens produced by chewing tea. Similar unpleasant symptoms have been observed among Congressmen addicted to the use of the cold infusion of this plant.

---

The Cleveland *Medical Gazette* reports a case in which a clergyman fractured his own rib while embracing one of his young female parishioners. This case will serve very well to point out a moral, but sounds a little fishy.

---

A parasitic disease has attacked cinchona trees in India. The growers are cutting them down, and the European market is flooded with lots of inferior bark.

In New York, there is one physician to every 500 inhabitants; in Philadelphia, one to 440, and in Chicago, one to every 365 inhabitants.

---

The vital statistics of Paris show that 28 per cent. of all children born are illegitimate. Of still-born, 35 per cent. are illegitimate.

---

The Pasteur Institute, of Paris, has already acquired an endowment amounting to 1,000,000 francs.

---

Cholera is rapidly increasing in Italy, and has invaded some of the cities and villages of Austria.

---

Vienna is to have a night medical service, modeled after that of Paris.

---

Dr. Agnew's works on Surgery are being translated into Japanese.

---

Small-Pox is epidemic in Buenos Ayres.

